# ‘You are not alone.’ An exploratory study on open-topic, guided collaborative reflection sessions during the General Practice placement

**DOI:** 10.1186/s12909-023-04756-6

**Published:** 2023-10-16

**Authors:** Chris W. Walinga, Pieter C. Barnhoorn, Geurt T.J.M. Essers, Sven P.C. Schaepkens, Anneke W.M. Kramer

**Affiliations:** 1 Department of Public Health and Primary Care, Leiden University Medical Centre, PO Box 9600, Leiden, 2300 RC The Netherlands; 2 Independent Researcher, Utrecht, The Netherlands; 3 Department of General Practice, Erasmus University Medical Centre, PO Box 2040, Rotterdam, 3000 CA The Netherlands

**Keywords:** Collaborative reflection, Clinical placements, Professional development, Medical education research, Undergraduate medical education

## Abstract

**Background:**

To support professional development of medical students faced with challenges of the clinical phase, collaborative reflection sessions (CRSs) are used to share and reflect on workplace experiences. Facilitation of CRSs seems essential to optimise learning and to provide important skills for lifelong learning as a professional. However, little is known about which workplace experiences students share in CRSs without advance guidance on specific topics, and how reflecting on these experiences contributes to students’ professional development. Therefore, we explored which workplace experiences students shared, what they learned from reflection on these experiences, and how they perceived the value of CRSs.

**Methods:**

We conducted an exploratory study among medical students (N = 99) during their General Practice placement. Students were invited to openly share workplace experiences, without pre-imposed instruction. A thematic analysis was performed on shared experiences and student learning gains. Students’ perceptions of CRSs were analysed using descriptive statistics.

**Results:**

All 99 students volunteered to fill out the questionnaire. We found four themes relating to students’ shared experiences: interactions with patients, complex patient care, diagnostic or therapeutic considerations, and dealing with collegial issues. Regarding students’ learning gains, we found 6 themes: learning from others or learning from sharing with others, learning about learning, communication skills, self-regulation, determination of position within the healthcare team, and importance of good documentation. Students indicated that they learned from reflection on their own and peer’s workplace experiences. Students valued the CRSs as a safe environment in which to share workplace experiences and helpful for their professional development.

**Conclusions:**

In the challenging General Practice placement, open-topic, guided CRSs provide a helpful and valued learning environment relevant to professional development and offer opportunities for vicarious learning among peers. CRSs may also be a valuable tool to incorporate into other placements.

**Supplementary Information:**

The online version contains supplementary material available at 10.1186/s12909-023-04756-6.

## Introduction

The process of professional development among medical students occurs throughout medical school, but it takes on a new dimension when they leave the pre-clinical classrooms and enter the clinical phase [1–3]. Here, students take on the physician role for the first time and are confronted with actual patients and their problems. These intense workplace experiences not only stimulate students to practice what they already know but also to deal with the inherent complexity and emotions that are frequently part of workplace experiences [4, 5]. Aside from the need to apply their existing knowledge and skills in practice, students also need to learn how to integrate these new experiences into their practice [6]. This adds an entirely new dimension to their learning process, challenging them to learn from work experiences.

Workplace experiences offer a great opportunity for professional growth. At the same time, students often feel stressed and thrown in at the deep end because of their new roles as physicians and the intensity of workplace experiences [1]. Learning effectively from workplace experiences may be hindered by stress in the transition to and during the clinical phase. Stimulating and training adequate reflective practice among medical students may enhance professional development in the clinical phase and enable self-directed lifelong learning [7]. Collaborative reflection sessions (CRSs) seem a particularly suitable way to enhance the reflective process due to the presence of both peers and a facilitator [8–16]. For facilitators of CRSs, it is essential to know how students can be optimally supported to fully profit from the learning potential of workplace experiences [6, 8, 17].

To promote reflection on workplace experiences, medical schools use several tools, e.g. portfolios, personal development plans, storytelling and written reflection assignments [6, 9, 17–19]. Gleeson et al. found that students preferred a group-based model of reflection over written reflection [20, 21]. Studies among medical students report the complex topics students reflect upon during small group meetings, such as critical incidents, complex patient cases, challenging communication and professional or ethical issues [5, 8, 11, 12]. These small group meetings are commonly based on preparatory written reflection assignments or predetermined themes. However, the key issues that arise from medical students sharing workplace experiences without advance guidance on specific topics are not yet fully clear. It could also be helpful to clarify the important factors within CRSs that contribute to the process of professional development. With this knowledge, we can further substantiate how the professional development of medical students during the clinical phase can be supported in CRSs. We therefore aimed to explore which topics from workplace experiences are shared with peers during CRSs in the General Practice (GP) placement, what students learn from reflecting on these issues and how they value the use of CRSs in the process of their professional development.

## Method

### Study design

To get more insight into CRSs, we conducted an exploratory study among medical students during their GP placement. As we were interested in the experiences of students and the meaning they give to these experiences, we chose a constructivist view for our research [22, 23].

### Setting

Medical school in the Netherlands consists of a 3-year preclinical Bachelor’s and a 3-year clinical Master’s program. We conducted our study at Leiden University Medical Centre (LUMC) during the 6-week GP placement which takes place in the second year of the Master’s program. With the GP placement, students complete compulsory placements before entering the third year’s electives. Every two weeks, a new group of medical students starts. In the second, fourth, and sixth weeks, a mandatory 3-hour small group session takes place. A facilitator, who is both an experienced GP and an experienced teacher, leads these small groups. Every small group session starts with approximately one hour of exchanging workplace experiences guided and structured by the facilitator. Without advance guidance on specific topics, students are invited to openly share workplace experiences that they find relevant. A CRS guide for facilitators is available, with a description of the steps to follow and pitfalls to avoid to ensure open discussion.

### Questionnaire

After the last small group session in the final week of the placement, we asked all students to fill out a paper questionnaire to evaluate the exchange of workplace experiences (see Supplementary Appendix 1). The questionnaire contained a combination of multiple-choice questions, open-ended and Likert scale queries. It aimed to collect demographic data, the number of workplace experiences shared by a student during the three CRSs, information about what workplace experiences were shared, what students learned from sharing the workplace experience, and what their perceptions were of the CRSs in relation to their professional development.

### Study population

For our study, we selected 11 consecutive groups that followed the GP placement between January and August 2017, containing 99 medical students. We invited all medical students in these groups to participate. With this number of participants, we expected to reach saturation. The number of participants for our study was within the range of other studies on this topic [7].

Participation was voluntary. We obtained informed consent and guaranteed confidentiality. The LUMC institutional ethical board determined that further ethical approval measures were unnecessary (reference P16.262).

### Data analysis

The paper questionnaires were digitalised by CW. All researchers had access to the data. Our dataset consists of quantitative and qualitative data. The quantitative data came from multiple-choice and Likert scale queries concerning age, gender, the number of shared workplace experiences, and the students’ perceptions of CRSs. The qualitative data arose from open-ended questions collecting the content of shared workplace experiences and learning gains.

#### General characteristics, Likert scale queries

We used IBM® SPSS® Statistics 25 for a descriptive analysis of the general characteristics of the study population, multiple-choice questions and Likert scale queries. We rejected questionnaires that were incomplete or unreadable.

#### Thematic analysis

We used ATLAS.ti® 7.5.18 and Microsoft® Excel® 2019 to support the qualitative data analysis. We performed a thematic analysis informed by the AMEE Guide on thematic analysis of qualitative data [24]. A detailed account of the analysis process is available in Supplementary Appendix 2. The team provided a range of perspectives and included experienced researchers and teachers from different backgrounds (General Practice, medical education, psychology, and philosophy) to ensure the confirmability, dependability, and credibility of the themes.

## Results

### General

All 99 students volunteered to fill out the questionnaire. Small group size varied from six to 19 students. Seventy-three students were female. The mean age of all students was 25 (standard deviation (SD) = 3.7 years). 27% of the students indicated that they had shared no workplace experiences during the three CRSs, 38% shared one workplace experience, and 35% shared two or more workplace experiences. Regarding the qualitative analysis of the open-ended questions, we excluded one questionnaire due to illegibility. Blank, unclear, or generally formulated answers were classified as missing data (37 responses concerning the content of shared workplace experiences and 22 responses concerning learning gains). Where applicable, we used multiple codes per response. For the statistical analysis of the Likert scale queries, we included 98 questionnaires.

### Shared workplace experiences

Workplace experiences formed the trigger for reflection during CRSs. We identified four main themes that students shared; *interactions with patients*; *complex patient care*; *diagnostic or therapeutic considerations*; and *dealing with collegial issues* (See Table 1).

For the students, *interactions with patients* during patient encounters often entailed demanding verbal or nonverbal communication, varying from difficulties in ‘finding out the request for help’ (J4) to situations where students had to deal with ‘aggression’ (A11, K1), ‘angry parents’ (B12), a ‘demanding patient’ (A8), a ‘concerned [patient]’ (C3) or a ‘[patient] who doesn’t cooperate’ (E5).

One case in this theme is worth mentioning, as it refers to the student’s own cognitions possibly contributing to make the interaction ‘complex’: ‘Thinking in boxes and its influence on consultation’ (A2).

The theme *complex patient care* concerned issues usually considered as complex and challenging care, for instance, caring for ‘frail elderly’ (B11), ‘end-of-life care’ (G2), or a ‘patient with suicidal intent’ (G1).

*The diagnostic or therapeutic considerations* students discussed were patient encounters in which they struggled with the ‘atypical’ (D15) presentation of medical problems or they reported that they treated a patient based on ‘gut feeling’ (D8). Some patient encounters led to unexpected outcomes, for example, when the patient with apparently non-alarming symptoms turned out later to have ‘appendicitis’ (F4).

Finally, the points raised by students related to *dealing with collegial issues* were workplace experiences about which they wondered ‘what to do if you disagree with the GP’ (H3), or struggled with the unprofessional behaviour of the other healthcare professionals they worked with, for example, when a supervising ‘GP was unavailable’ (D19).

**Table 1:** Shared workplace experiences

Theme	Example
Interactions with patients	‘A patient with macroscopic haematuria was very concerned. To what extent do you go along with these concerns, and how do you express this?’ (C3)
Complex patient care	‘A patient with suicidal intent that I had seen alone, with a possible underlying personality disorder’ (G1)
Diagnostic or therapeutic considerations	‘Deciding to refer someone [to the emergency room] with atypical chest pain’ (D15)
Dealing with collegial issues	‘A man with Parkinson’s did not dare to walk, and nurses yelled at him not to cry and just walk’ (F1)

### Student learning gains

Students reported learning related to both sharing their own workplace experiences, and hearing the workplace experiences of their peers. Concerning student learning gains, we distilled six main themes: *learning from others or learning from sharing with others*, *learning about learning*, *communication skills*, *self-regulation*, *determination of position within the healthcare team* and *importance of good documentation* (See Table 2).

The first theme *learning from others or learning from sharing with others*, was frequently reported by students. Students expressed how the presence of others (peers or supervisors) was valuable to: offer a *different perspective* on a workplace experience; *get feedback on their own actions*; mentioning the *value of follow-up discussion with the supervisor* in the workplace; or gain *recognition or insight into the perception of others* when they noticed, for example, the feelings, struggles or uncertainties of peer students. The action of sharing itself was also helpful for students, offering an opportunity for *venting*.

With regard to the second theme, *learning about learning*, students indicated that they discovered several aspects of learning in the clinical setting: the value of *evaluation* and *learning on-the-job*, acknowledging that *making mistakes is part of the learning process*, weighing up *protocol versus own insight* and the value of *self-reflection*.

Thirdly, students also reported that they gained tools to enhance *communication skills* in the workplace with patients and supervisors, for example, ‘metacommunication’ (A3, A11) or ‘how to deal with difficult family members’ (I5).

The fourth theme, *self-regulation*, including *setting boundaries, dealing with your own emotions, dealing with independence*, and *standing up for yourself*, shows how reflection during CRSs helped students make explicit how to remain balanced in the challenging clinical learning environment.

The fifth theme, *determination of position within the healthcare team*, indicates how CRSs helped students clarify their role or skills in the workplace, facilitating collaboration with healthcare professionals.

Finally, the sixth theme, *importance of good documentation*, refers to the value students assigned to good documentation related to patient encounters.

**Table 2:** Student learning gains

Theme	Sub-theme	Example
Learning from others or learning from sharing with others	Different perspective Getting feedback on own actions Recognition or insight into the perception of others Value of follow-up discussion with the supervisor Venting	‘That you are not alone with particular frustrations or insecurities’ (C5)
Learning about learning	Evaluation Learning on-the-job Making mistakes is part of the learning process Protocol versus own insight Self-reflection	‘Pausing for a moment, evaluating and reflecting on a conflict really works. Whether it is on paper or with peers. I would like to carry on doing this for a complex case’ (C1)
Communication skills	With a patient With a supervisor Unclear to whom communication relates	‘How to ask questions to help someone get an answer without you answering yourself’ (A4)
Self-regulation	Dealing with independence Dealing with your own emotions Setting boundaries Standing up for yourself	‘Stay true with yourself, and be aware of your limits’ (D2)
Determination of position within the healthcare team	No subthemes	‘Role and obligations as a student within the team’ (D9)
Importance of good documentation	No subthemes	‘Good documentation so that you can look back afterwards and explain why you made certain decisions’ (B6)

### Student perceptions of CRSs

Students perceived the CRSs as safe and essential for their professional development as physicians and felt better able to deal with similar situations next time around (see Table 3).

**Table 3:** Student perceptions of CRSs (5-point Likert scale; 1: strongly disagree, 5: strongly agree)

Statement	Mean (SD)
I felt safe sharing workplace experiences	4.4 (0.6)
Sharing workplace experiences is essential right now in my training	4.0 (0.7)
Sharing workplace experiences is essential to my development as a physician	4.1 (0.7)
After discussing workplace experiences (my own or that of a peer), I felt more skilled the next time I encountered a similar situation.	3.8 (0.7)

## Discussion

This study examined the use of CRSs to support undergraduate medical students’ professional development during their GP placement. We aimed to explore which issues related to workplace experiences students shared, what learning gains they reported after reflection on these issues, and how students valued CRSs.

### Shared workplace experiences

CRSs provide opportunities to share any workplace experience without pre-imposed instruction, for instance, experiences of unprofessional behaviour or critical incidents. Thus, we were able to gain a broad view of the essential issues from which students learn in the clinical phase of their training. Students discussed issues arising from patient encounters and situations with co-workers and supervisors. Those issues were often complex or out of the ordinary, for example, when students had to deal with a complicated patient or an atypical presentation or progression of a medical condition. Our findings provide further substantiation that CRSs are a valuable educational tool to foster reflection on issues relevant to the professional development of students. Our study also indicates that an open invitation to share workplace experiences, without, for example, a predetermined theme or preparatory assignment, is sufficient to motivate students to reflect on issues that are important for their professional development. The additional advantage of this open invitation is that students decide for themselves what they want to discuss. The need to discuss complex issues during the CRSs emphasises the challenges students experience in the clinical phase. The clinical phase makes students realise that they still have a lot to learn in applying knowledge and skills [1]. The safe classroom environment they encounter in the preclinical setting, with its well delineated patient cases and clear frameworks, has now disappeared. While dealing with, for example, aggressive or terminally ill patients can be prepared for in theory, or in a simulated setting, it can only be experienced and learned to its fullest extent in practice [25]. Sharing workplace experiences in an open, guided CRS provides a suitable context for experiential learning.

### Student learning gains

The learning gains of reflection describe how CRSs may contribute to both the process and content of professional development. The process concerns aspects of ‘becoming’ a physician, whereas the content focuses more on ‘doing the work of a physician’ [26].

The theme *learning from others or learning from sharing with others* not only reflects how students are helped by others in reflective practice but also that sharing itself is valuable. In essence, it is the recognition of these valuable aspects of sharing that allows students to learn what is described in the other themes. During CRSs, both student peers and facilitators are present. This provides an environment that, among other things, shows students different perspectives and gives them opportunities to vent. These aspects illustrate the potential that the supportive context provided by CRSs gives for reflection on workplace experiences. Furthermore, the theme *learning about learning* shows how different aspects of managing learning were made explicit during CRSs and thus also contributed to the process of professional development, for example, through recognition of the role of evaluation, making mistakes, or self-reflection for learning.

Several studies underline the value of having peers present during reflection in small groups. The presence of peers offers opportunities for recognition of similar experiences [10, 12], a reality check [10], strategies for coping [8, 12], reducing stress and encouraging professional development [7, 12]. Social interaction between peers is essential to the process of learning and professional identity formation [11, 26–28]. Considering that the transition to the clinical phase is a vulnerable period for professional identity formation, Kay et al. underline the importance of integrating purposeful educational interventions [29]. In essence, each new clinical placement can be seen as a transition from theory to a new speciality-specific practice, and even more in the GP setting because of the one-to-one relationship with the supervising GP and the absence of peers [19]. Social interaction with peers and facilitators during CRSs can be part of such a meaningful intervention and thus support professional identity formation. Our study adds to existing literature indicating that hearing others’ workplace experiences and engaging in a dialogue about those experiences during CRSs also generates learning gain [12]. CRSs offer opportunities to bring together valuable workplace experiences and learn from them without the students having actually experienced every situation themselves, echoing the concept of vicarious learning [30]. Furthermore, CRSs allow students to learn to reflect on these workplace experiences from different perspectives. Given the diversity of medical practice and the limited duration of clinical placements, CRSs may represent a welcome addition to workplace learning, enabling exposure to more workplace experiences than any individual student can experience during medical school.

Managing learning in the clinical phase is also challenging for students and requires new skills. Peers play an essential role in managing learning, including through social comparison [31, 32]. Our study suggests that CRSs support students in developing these new learning skills in the clinical phase of training.

We distilled four themes concerning how CRSs contributed to the content of professional development: *communication skills*, *self-regulation*, *determination of position within the healthcare team*, and *importance of good documentation*. These themes point to several essential skills needed to act competently in clinical practice and resonate with several competencies within the CanMEDS competency Framework [33], namely Communicator, Professional, Collaborator, and Leader. Our study indicates that using CRSs as an educational intervention can contribute to competence development as a part of the professional development of students in the clinical phase. Reflection on workplace experiences in small groups with the support of peers and a facilitator is known to be an effective practice for supporting clinical competence [34, 35]. This may happen in a number of ways, including helping students face complex patient communication, developing skills in effective teambuilding and remaining balanced in the challenging clinical environment [5, 10].

### Student perceptions of CRSs

We found that students valued the CRSs as safe and helpful for their development into medical professionals. CRS offered the possibility of a low threshold for sharing workplace experiences and hearing authentic experiences from others in real-time. This may have contributed to students’ perceived importance of CRSs. Our findings resonate with other studies reporting that reflection in small groups is much appreciated by students, helpful in reflecting on workplace experiences during clinical placements and contributes to professional development [8, 10, 12, 20]. More importantly, small groups offer a safe environment in which to discuss unspoken topics with others who are able to fully appreciate the context of the situation at hand [8].

### Strengths and limitations

Our study had a high response rate, which may indicate that students were enthusiastic about this form of reflection and for that reason were motivated to participate. Performing a thematic analysis, we inductively analysed these data using the different perspectives of an interdisciplinary team to ensure confirmability, dependability, and credibility.

However, our study had several limitations. It was performed at a single institution and was limited to the GP placement. This may reduce the transferability to other clinical placements and different medical curricula. Our survey used open-ended questions. This often resulted in short answers that were open to multiple interpretations. While we gained many insights from a significant number of students, we could not probe these short answers for clarification. Applied to exploring learning gains, the use of a questionnaire and asking only about the most important learning gains may have resulted in missing aspects of learning that were less prominent or acquired along the way, for example, basic medical knowledge or skills. We collected our data in 2017. In the intervening period, the COVID-19 pandemic has occurred. During the pandemic, general GP care was compromised, and university education mainly took place online. GP care in The Netherlands at this moment is largely comparable to before the pandemic. At our institution, CRSs are organised in the same way now as they were when the data reported in this manuscript were collected, and no revisions have been made to the curriculum. Lastly, answers may be subject to recall bias because we asked students to fill out our questionnaire after three CRSs, spread over a four-week period at the end of the GP placement.

### Further research

Our exploratory study invites further in-depth research to understand what students grapple with during the clinical phase of their training, and how collaborative reflection can support them. Further research should focus on; why specific issues are essential for students in transition to, and during, the clinical phase; what goals students had in mind when sharing their particular workplace experience and how sharing during CRSs contributed to achieving those goals; and how collaborative reflection during CRSs contributes to competence. In addition, further elaboration is needed on how CRSs relate to other forms of reflection in supporting professional development such as personal development plans or reflective writing assignments.

## Conclusions

Our findings indicate that CRSs may be a valuable educational tool to foster reflection on relevant issues for medical students when faced with the challenges that arise in the GP placement. Open-topic, guided CRSs provide low-threshold opportunities to share any workplace experience at the appropriate moment, which may also be valuable to incorporate into other placements. The safe learning environment and presence of others during CRSs may increase the learning gains of reflection. As a result, CRSs offer opportunities to support professional development across the full breadth of the profession.

### Electronic supplementary material

Below is the link to the electronic supplementary material.

Supplementary Material 1

Supplementary Material 2

## Data Availability

The datasets used and/or analysed during the current study are available from the corresponding author on reasonable request.

## Abbreviations

CRSs: Collaborative reflection sessions
GP: General Practice
SD: Standard deviation
