# Supplementary material for: ‘You are not alone.’ An exploratory study on open-topic, guided collaborative reflection sessions during the General Practice placement
Source: BMC Med Educ. 2023 Oct 16;23:769. doi: 10.1186/s12909-023-04756-6 (PMC10577966; doi:10.1186/s12909-023-04756-6)
Supplement: Supplementary file 1 — Supplementary Material 1 [file 12909_2023_4756_MOESM1_ESM.docx]

# Supplementary Appendix 1: Questionnaire

1. What is your gender? (Multiple choice: male, female)
2. What is your age? (Open-ended question)
3. How many times did you share your workplace experiences during the small group sessions? (Multiple choice: 0, 1, 2, 3, 4, more than four times)
4. Please describe concisely any workplace experiences you shared. (Open-ended question)
5. What is the most important learning gain you have taken from sharing your own or a peer's workplace experience? (Open-ended question)
6. Does your most important learning gain relate to your own workplace experience, the workplace experience of a fellow student, or both? (Multiple-choice: own, peer, both)
7. Can you indicate to what extent the following statements apply to you? (Five-point Likert scale: strongly agree – agree – neither agree nor disagree – disagree – strongly disagree)
   1. I felt safe sharing workplace experiences.
   2. Sharing workplace experiences is essential right now in my training.
   3. Sharing workplace experiences is essential to my development as a physician.
   4. After discussing workplace experiences (my own or that of a peer), I felt more skilled the next time I encountered a similar situation.
