# Supplementary material for: ‘You are not alone.’ An exploratory study on open-topic, guided collaborative reflection sessions during the General Practice placement
Source: BMC Med Educ. 2023 Oct 16;23:769. doi: 10.1186/s12909-023-04756-6 (PMC10577966; doi:10.1186/s12909-023-04756-6)
Supplement: Supplementary file 2 — Supplementary Material 2 [file 12909_2023_4756_MOESM2_ESM.docx]

# Supplementary Appendix 2: Description of the process of thematic analysis

Step 1: Familiarisation with the data. CW, PB, and SS read all questionnaires independently. The unreadable or incomplete answers were discussed, leading to a consensus about in- or exclusion for analysis.

Step 2: Generating Initial Codes. CW, PB, and SS individually coded the open questions of the whole dataset using an inductive approach. The open questions were coded separately, but in some cases, information from open questions was used together to better interpret the data. Where this occurred, it was documented. CW, PB, and SS compared and discussed the individually assigned codes per participant, which led to a coding scheme with three codes regarding the Shared workplace experiences and five codes regarding the Learning gains. Subsequently, CW, PB and SS individually applied the codes from this coding scheme to the entire data set. The assignment of multiple codes per participant was permitted. After this, CW, PB, and SS again compared the individually assigned codes and discussed where they differed, leading to consensus. When no consensus was reached, the data were classified as missing.

Step 3: Searching for themes. CW, PB, and SS separately searched for themes and subthemes. After several internal team discussions, CW, PB, and SS formulated an initial theme map. The initial theme map was presented to GE. GE checked whether he agreed with the themes found and whether he thought they represented the entire data set. This led to a proposal to adjust the initial theme map, to which CW, PB, and SS agreed.

Step 4: Reviewing themes. We discussed this theme map with the whole team (AK, CW, GE, PB, SS) to ensure that each theme had enough supportive data and to determine its relationship to the research question. The themes found within the Shared workplace experiences turned out not to provide a good answer to the research question: this led to a redefinition of these themes. In the themes found within the Learning gains, some themes could be combined. The theme 'Learning from or sharing with others’ was changed to ‘Learning from others, or learning from sharing with others’. CW re-read the complete dataset to finalise this step, ensuring the themes represented all data.

Step 5: Defining and naming themes. We reached a consensus, which led to the definition and naming of the themes found. CW selected data extracts that could best illustrate those themes in the final manuscript.

Step 6: Producing the report/manuscript: CW drafted the manuscript, providing a narrative of why the themes and the interpretation of the themes were relevant to the research question, presenting relevant data extracts, and discussing implications. All authors critically reviewed and commented on the manuscript. After several versions, this led to the final manuscript.
